# Revealing the nature of morphological changes in carbon nanotube-polymer saturable absorber under high-power laser irradiation

**DOI:** 10.1038/s41598-018-24734-z

**Published:** 2018-05-10

**Authors:** Maria Chernysheva, Mohammed Al Araimi, Graham A. Rance, Nicola J. Weston, Baogui Shi, Sayah Saied, John L. Sullivan, Nicholas Marsh, Aleksey Rozhin

**Affiliations:** 1 Nanotechnology Research Group and Aston Institute of Photonic Technologies, Aston University, Birmingham, B4 7ET UK; 2 Al Musanna College of Technology, Muladdah, Al Musanna, Sultanate of Oman, Muladdah, Oman; 3 Nanoscale and Microscale Research Centre (nmRC), Cripps South, University of Nottingham, University Park, Nottingham, NG7 2RD UK; 4 Surface Science Research Group, Aston University, Birmingham, B4 7ET UK; 5 Keyence(UK), Ltd, Avebury House, 219-225 Avebury Boulevard, Milton Keynes, MK9 1AU UK

## Abstract

Composites of single-walled carbon nanotubes (SWNTs) and water-soluble polymers (WSP) are the focus of significant worldwide research due to a number of applications in biotechnology and photonics, particularly for ultrashort pulse generation. Despite the unique possibility of constructing non-linear optical SWNT-WSP composites with controlled optical properties, their thermal degradation threshold and limit of operational power remain unexplored. In this study, we discover the nature of the SWNT-polyvinyl alcohol (PVA) film thermal degradation and evaluate the modification of the composite properties under continuous high-power ultrashort pulse laser operation. Using high-precision optical microscopy and micro-Raman spectroscopy, we have examined SWNT-PVA films before and after continuous laser radiation exposure (up to 40 hours) with a maximum optical fluence of 2.3 mJ·cm^−2^. We demonstrate that high-intensity laser radiation results in measurable changes in the composition and morphology of the SWNT-PVA film due to efficient heat transfer from SWNTs to the polymer matrix. The saturable absorber modification does not affect the laser operational performance. We anticipate our work to be a starting point for more sophisticated research aimed at the enhancement of SWNT-PVA films fabrication for their operation as reliable saturable absorbers in high-power ultrafast lasers.

## Introduction

The first experimental observation of strong nonlinear optical and electro-optical properties of SWNTs, and later of graphene and transition metal dichalcogenides (TMDs), caused a new era in photonics through the demonstration of nanomaterial based Saturable Absorbers (SA) for laser mode-locking^1–5^ and noise suppression filters^6,7^. The attractiveness of nanomaterials-based SAs is primarily caused by the ultrafast excited state carrier dynamics^8^ and high third-order optical nonlinearity^2,9^, but also because of the simplicity of fabrication compared to the more commonly used saturable absorbers, e.g. semiconductor saturable absorber mirror (SESAM)^10^. Specifically, semiconductor SWNTs are direct-bandgap materials with an energy gap dependent on the particular diameter and chirality of the SWNT. This allows broad operation wavelengths for SWNT-based SAs from about 750 nm to beyond 2 *μ*m^8,9,11,12^, which was confirmed by the demonstration of a large number of SWNT mode-locked lasers in the mentioned wavelengths range^1,2,13–18^.

Although many different methods have been proposed for the production of nanomaterials based SAs, such as spaying or direct growth on optical parts, nanomaterial-polymer composite SAs have received the most widespread attention because of the simplicity of integration into optical systems. Moreover, a large variety of polymers, both soluble in water (e.g. PVA, carboxymetylcellulose (CMC)) and organic solvents (e.g. polymethylmethacrylate, polycarbonate, polystyrene) have been used as a matrix for the fabrication of nanomaterial-based SA with SWNTs of particular interest^2,19^. Despite the low environmental stability, WSP-based SAs (mostly PVA and CMC) have dominated so far^2,20–22^, because they enable control of resulting parameters of the nanocomposite, such as polymer film thickness and SWNT concentration, alignment^23^, distribution and bundle size^24^ of SWNTs within the matrix. These in turn determine the saturable absorption properties of the SWNT composites including recovery time, saturation intensity, modulation depth, and non-saturable loss^23,24^. Additionally, PVA has gained widespread use among WSPs, since the resulting composites are mechanically strong and have smooth surfaces^2^.

For a longtime, SWNT-WSP (typically CMC and PVA) SAs have been reported to suffer from a low thermal damage threshold and, therefore, demonstration of high power generation utilising SWNT-PVA SAs appeared to be challenging^17,25^. This is because high-intensity optical pulses propagating through the laser cavity can be destructive for laser components and material SAs with polymers, in particular. The diversity of integration configurations of SWNT-based SAs into laser cavities have been demonstrated. These approaches are usually based on the interaction of SWNTs with the evanescent field of the propagating core mode in the optical fibre, utilising specially designed constructs, such as D-shaped^26^, tapered^27^ or microstructured optical fibres^28^. However, such a special optical fibre design induces unavoidable polarisation sensitivity, high nonlinearity, large group velocity dispersion in the laser cavity^29^, or additional losses during splicing with other types of fibres, *e.g*. standard single-mode or active fibre. Despite the prevailing opinion that high intracavity optical power induces thermal damage of carbon nanotubes, our previous works have demonstrated the great potential of SWNT-PVA films, sandwiched between two optical ferrules. We have shown an output power reaching 300 mW and a pulse energy of 10 nJ, parameters that are usually not reachable for generation directly from a laser with SWNT SAs without subsequent amplification^17,30^.

This study aims to unfix all of the traditional notions and extend the understanding of the thermal degradation and damage threshold of a conventional ferrule-type SAs, sandwiching a thin polymeric film containing homogeneously dispersed SWNTs in a high-power passively mode-locked Erbium-doped fiber laser (EDFL) with output parameters: 550-fs pulse duration, 18.5 MHz repetition rate, average output power of 50 mW and peak power of 4.78 kW. The SWNT-PVA film, operating as an SA, demonstrates a high thermal degradation threshold. For the first time, we have investigated the SWNT-PVA film after continuous high-power irradiation in an EDFL over time intervals from 10 min to 40 hours under laboratory conditions. After the initial modification of the SWNT-PVA composite under high-intensity internal inter-cavity radiation, the material is modified into a stable SA. This was confirmed by investigation of the composite’s surface and topological properties by the methods of micro-Raman spectroscopy and high-resolution digital optical microscopy.

## Results

### SWNT characterisation

The assembly and structural and functional characterisation of the SWNT-PVA films is described in detail in the *Methods* section. In brief, stable aqueous suspensions of SWNTs, made by the high-pressure carbon monoxide (HiPCO) process, were prepared by ultrasonic-assisted homogenization in the presence of the surfactant sodium dodecylbenzene sulfonate (SDBS), followed by the addition of powdered polyvinyl alcohol (PVA) and subsequent evaporation of the water in a desiccator to yield a freestanding thin SWNT-PVA film (∼50 *μ*m). The SWNTs feature diameters ranging from ∼0.8–1.3 nm and possess a high percentage of semiconductor SWNTs^11,31^.

**Figure 1:**
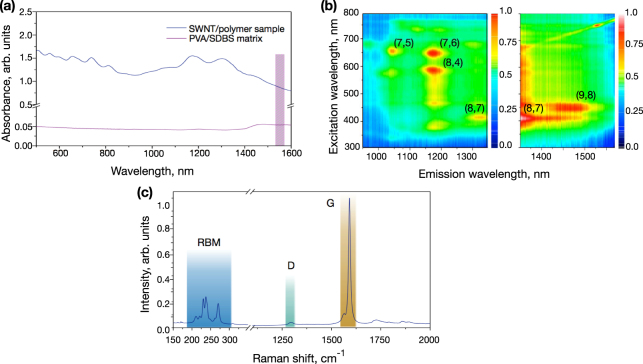
(**a**) UV-visible-NIR absorbance spectrum of the SWNT-PVA composite film. The contribution from the PVA matrix has been subtracted. The coloured bar at 1530–1560 nm indicates the laser operation band. (**b**) Normalised photoluminescence (PL) map of the SWNT-PVA film. (**c**) First order Raman spectrum of the SWNT-PVA composite film.

Figure 1(a) shows the optical absorption spectrum of the SWNT-PVA film with the subtracted background of PVA (blue plot). The optical absorption spectrum has been measured using a Lambda 1050 UV-NIR spectrometer (Perkin Elmer). Three main absorption regions are presented in the spectrum due to SWNT interband transitions. The spectral features from 1600 to 1100 nm and from 900 to 700 nm correspond to the E_11_ and E_22_ excitonic transitions of semiconductor SWNTs (s-SWNTs), respectively^11,32^. The spectral feature below 550 nm is due to the M_11_ transition of metallic SWNTs (m-SWNTs)^11^. The high-intensity peaks appeared in the NIR region are caused by the absorption of s-SWNTs with diameters ranging from 0.8 to 1.3 nm^33^. Although the intensities of these peaks are centred in the spectral range of 1175 and 1300 nm, respectively, the spectrum also exhibits strong optical density about 0.85 at 1560 nm due to the presence of tube diameters ∼1.2 nm.

The 2D normalised photoluminescence (PL) map of SWNT-PVA film as a function of emission (λ_11_) and excitation (2λ_22_) wavelengths is shown in Fig. 1(b). The map has four bright PL features in the range of 950–1350 nm excitations, assigned to the emission from the first interbands (E_11_) of (7, 5), (7, 6), (8, 4) and (8, 7) chiralities of SWNTs, followed by the photoexcitation to the second interband (E_22_) (left map). The right part of the plot shows an enhanced view of the PL region from 1350 to 1570 nm. This region is overshadowed by high-intensity peaks of the main chiralities with emission around 1200 nm. Thus, for detailed examination it has been normalised separately. The right plot feature PL peak of the (9, 8) chirality.

Figure 1(c) shows the first order Raman spectrum of the SWNT-PVA films, mounted onto glass slides, acquired using a 785 nm (1.58 eV) laser. It is important to note that the energy of the laser matches the E_22_ transition in a subset of s-SWNTs (as shown in Fig. 1a) according to the Kataura plot^11^. This set of SWNTs are therefore in resonance with the excitation laser and appear preferentially in the resultant spectrum, masking the contribution from both SWNTs that do not satisfy the resonance condition and the host polymer matrix itself. The key features in the first order Raman spectra of SWNTs are the radial breathing modes (RBMs, 150–300 cm^−1^) and the D (disorder, ∼1350 cm^−1^) and G (graphite, ∼1590 cm^−1^) bands^12^. The RBMs correspond to low-frequency vibrations of carbon atoms in the radial direction and are highly sensitive to the diameter and chirality of SWNTs. There are seven observable RBMs in the spectrum of the SWNT-PVA film, with three dominant modes at 230.7, 237.4 and 270.0 cm^−1^. The relationship between the frequency of the RBMs *ω*_*RBM*_ and the nanotube diameter *d* can be described as *ω*_*RBM*_ = 234/d + 10 and thus the three dominant RBMs could correspond to s-SWNTs with diameters of 1.06, 1.03 and 0.90 nm, respectively. A lower intensity RBM at 211 cm^−1^ corresponds to CNT with diameters of 1.17 nm, which are essential for mode-lock initiation at ∼1560 nm. This is in consistency with the (9, 8) chirality, which represents the dominant feature in PL map (Fig. 1b). Further evidence of the semiconducting nature of the SWNTs in resonance with the excitation laser is afforded from the lineshape of the G band – a stretching vibration of E_2*g*_ symmetry present in all graphitic lattices. The G band is described by a simple Lorentzian function, which is split into two bands, a smaller mode (G^−^) at ∼1565 cm^−1^ and a larger mode (G^+^) at ∼1590 cm^−1^, corresponding to vibrations either perpendicular or parallel to the carbon nanotube axis, respectively. The D band is a ring-breathing mode of A_1*g*_ symmetry, requiring a defect (e.g. hetero-atoms, vacancies, Stone-Wales defects, kinks, impurities, *etc*.) for its activation, and is often used to diagnose the structural purity of graphitic nanostructures. Of note, the intensity ratio of D and G bands (I_*D*_:I_*G*_) in the SWNT-PVA film is 0.04, indicative of a high structural purity of the graphitic nanotube sidewalls.

### High power ultrafast laser radiation exposure

**Figure 2:**
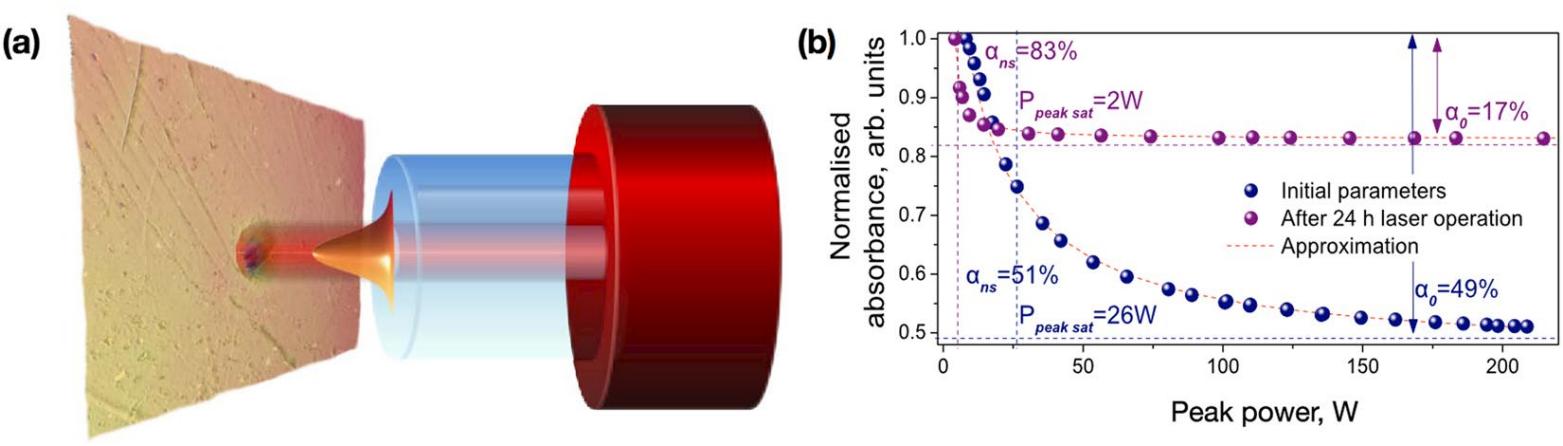
(**a**) Schematic representation of the SWNT-PVA film exposure to high-power fibre laser radiation. (**b**) Power-dependent measurements of the SWNT-PVA film before (blue) and after (purple) laser operation at the maximum available pump power P_*pump*_ = 600 mW. The circles are the experimental data and the dashed curves represent an analytical fit of the data.

In our experiments, we used a specially developed Erbium-doped SWNT mode-locked fibre laser, described in section *Methods*, with the SWNT-PVA film acting as a SA. Figure 2(a) shows a schematic representation of the fibre laser radiation exposure to the SWNT-PVA film. The film is placed between two optical connectors so that it adjoins the optical fibres. The optical fibre used to create the SA is standard single mode fibre SMF-28, which permits the assumption that the output beam has a Gaussian profile. The Gaussian beam is typically characterised regarding mode field diameter, which is a beam diameter at the normalised intensity level of 1/e^2^. As it can be seen from Fig. 2(a), the diameter of the laser affected area of the SWNT-PVA film is slightly less than the mode field diameter of 10.5 *μ*m.

While measuring the power-dependent optical properties, the power of the probe radiating on the parent SWNT-PVA film was increased from 0.1 to 3 mW. The blue plot in Fig. 2(b) shows the measured power dependent absorption and modulation depth of the SWNT-PVA film before the experiments on high power laser generation. The measured modulation depth and non-saturable losses of the SWNT-PVA film SA are *α*_0_ = 54% and *α*_*ns*_ = 46%, respectively. Such a high value of *α*_0_ is particularly beneficial for stable mode-locking initiation and operation^2^. The saturation intensity and saturation peak power, which are the optical pulse intensity and pulse peak power required for reduction of the absorption coefficient to half the initial value, were measured as I_*sat*_ = 58.8 MW · cm^2^, P_*peaksat*_ = 20 W, correspondingly. The growth of samples transmission T is 47%.

To understand the nature and characterise the transformation of both the SWNTs and the hosting PVA polymer matrix, several identical SWNT-PVA films underwent high-power ultrashort pulse laser action at different powers (from 15 to 50 mW, which correspond to pulse energies in the range 0.9–2.63 nJ) and exposure times (10 minutes to 40 hours). The laser has been set at a pump power of 600 mW, achieving the output pulse energy of 2.63 nJ, with a corresponding optical fluence of 2.3 mJ · cm^−2^. Since the output coupler in the laser ring cavity had 50:50 splitting ratio, we can assume that the laser irradiation launched on the SWNT sample was of the same energy value. In a series of control measurements, samples of pure PVA and PVA with SDBS – the surfactant used for stabilisation of the SWNT suspension during materials processing – were ablated for 2 hours with the same optical fluence.

The power-dependent properties of the SWNT-PVA film after laser radiation exposure have been analysed. For this, we have taken the as-operated SWNT-based SAs, i.e. optical fibre pigtails with a squeezed SWNT-PVA film in between them, and incorporated it into the measurement setup as described in the Methods section. The purple plot in Fig. 2(b) shows the alteration of the power-dependent characteristics after 24 hours of continuous laser exposure. The modulation depth *α*_0_ decreased to 17%, whereas the saturation intensity *I*_*sat*_ dropped to 2 W/cm^2^. The non-saturable losses *α*_*ns*_ increased to 83%. The observed changes in the composites saturable absorption properties are apparently caused by the surface properties of the modified composite, which can lead to both additional reflection and scattering on roughness. In addition, laser modification of the near-surface layer can form SWNT bundles of large diameters and, as a result, can cause additional non-saturable losses due to scattering. Despite the assumptive surface damage and change in the polymer morphology of the SWNT-PVA film, its modulation depth is still of sufficient value to efficiently initiate self-starting mode locking. When the measured SWNT SA was returned into the ultrafast laser cavity, the generation performance featured unchanged output parameters after several on-off cycles with the help of slight PC tuning.

#### Optical microscopy analysis of the SWNT-PVA films after laser radiation exposure

**Figure 3:**
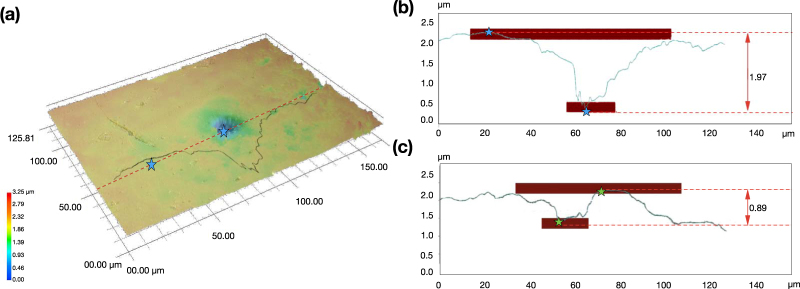
(**a**) Optical micrographs and (**b**) cross-sectional profile of the laser exposed areas of the SWNT-PVA film after 40 hours of continuous laser radiation exposure with an optical fluence of 2.3 mJ·cm^−2^; (**c**) Cross-section profile of SWNT-PVA film after 10 min laser exposure with 0.9 mJ · cm^−2^.

The irradiated SWNT-PVA films undergo complex transformations during ultrashort pulse laser ablation. Microscopic images of the SWNT-PVA films were acquired after high power radiation exposure to inspect the surface modification. Figure 3 shows a three-dimensional optical micrograph of the top surface (a) and a cross-sectional profile of the SWNT-PVA film (b) after 40 hours and 10 min (c) of laser operation with 2.3 mJ · cm^−2^ optical fluence launched onto the film.

Three-dimensional depth profile measurements have been performed with Z-axis resolution of 0.1 *μ*m. The image in Fig. 3(a) demonstrates a part of the samples with length and width of 125.81 and 170 *μ*m, respectively. A single clear spot of degradation in the composite film has been observed as a crater with a diameter of ∼14 *μ*m and a depth of 1.95–2.5 *μ*m (∼10 *μ*m at the base of the crater). As stated previously, the sample thickness is ∼50 *μ*m, which means that only the top surface of the film is affected subsequent to the laser exposure.

A similar spot has been observed after 10 min laser operation with optical fluence 0.9 mJ · cm^−2^ launched on the film (Fig. 3c). The laser penetration depth reaches 0.89 *μ*m, and the width remains at 9.5 *μ*m. It is important to note that the crater diameter remains in the range 9.5–10 *μ*m regardless of the radiation exposure time and optical fluence (see Table 1). A crater formation at low power laser radiation during short-term operation suggests that the film modification occurs at the very beginning of the high power operation. Further investigation of various times of laser exposure at the maximum available optical fluence of 2.3 mJ · cm^−2^ demonstrates that the crater depth after 2 hours of continuous laser operation stops increasing and levels off at 2–2.36 *μ*m. Such film stabilisation enables laser operation in the long-term stable mode-locking regime (as demonstrated in section *Methods*, in Fig. 5) and without breaking to CW or Q-switching. At the same time a rapid increase of intracavity optical fluence, without simultaneous laser regime stabilisation using polarisation controllers, results in deep crater formation (6.1 *μ*m depth) after a 10 min laser exposure. Table 1 summarises the laser irradiation condition and the geometry of laser exposed areas of SWNT-PVA films.

**Table 1:** Summary of crater geometry formed on SWNT-PVA film after laser exposure.

Exposure time	Optical fluence, mJ · cm^−2^	Crater depth, *μ*m	Crater diameter *μ*m
10 min	0.9	0.89	9.5
10 min	2.3	6.1	8
2 hours	2.3	2.3	7
4 hours	2.3	2.36	9
6 hours	2.3	1.5	15
24 hours	2.3	2	9.5
40 hours	2.3	1.97	10

**Figure 4:**
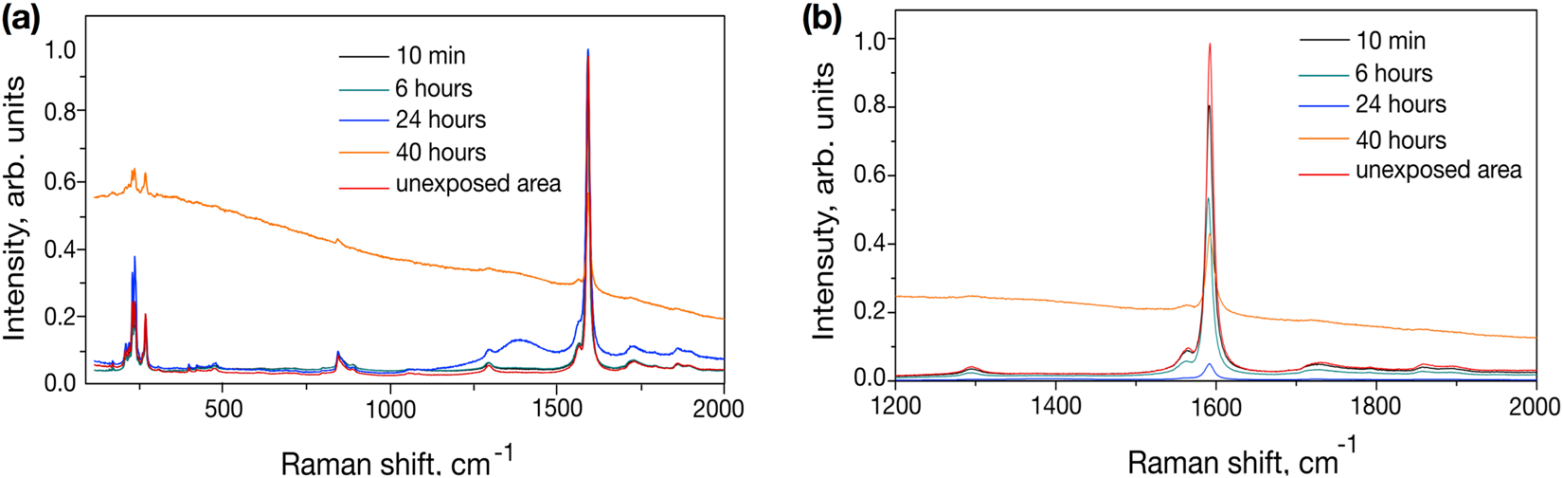
Raman spectra, collected from exposed and unaffected areas of SWNT-PVA films after 10 min continuous laser radiation exposure with an optical fluence of 0.9 mJ · cm^−2^, and 6 hours, 24 hours, and 40 hours under optical fluence of 2.3 mJ · cm^−2^ (**a**) Spectra normalised to the intensity of the G band; (**b**) Zoomed on G band, normalised to acquisition time and conditions at the bottom of the crater (laser exposed area) and on the top unaffected surface.

**Figure 5:**
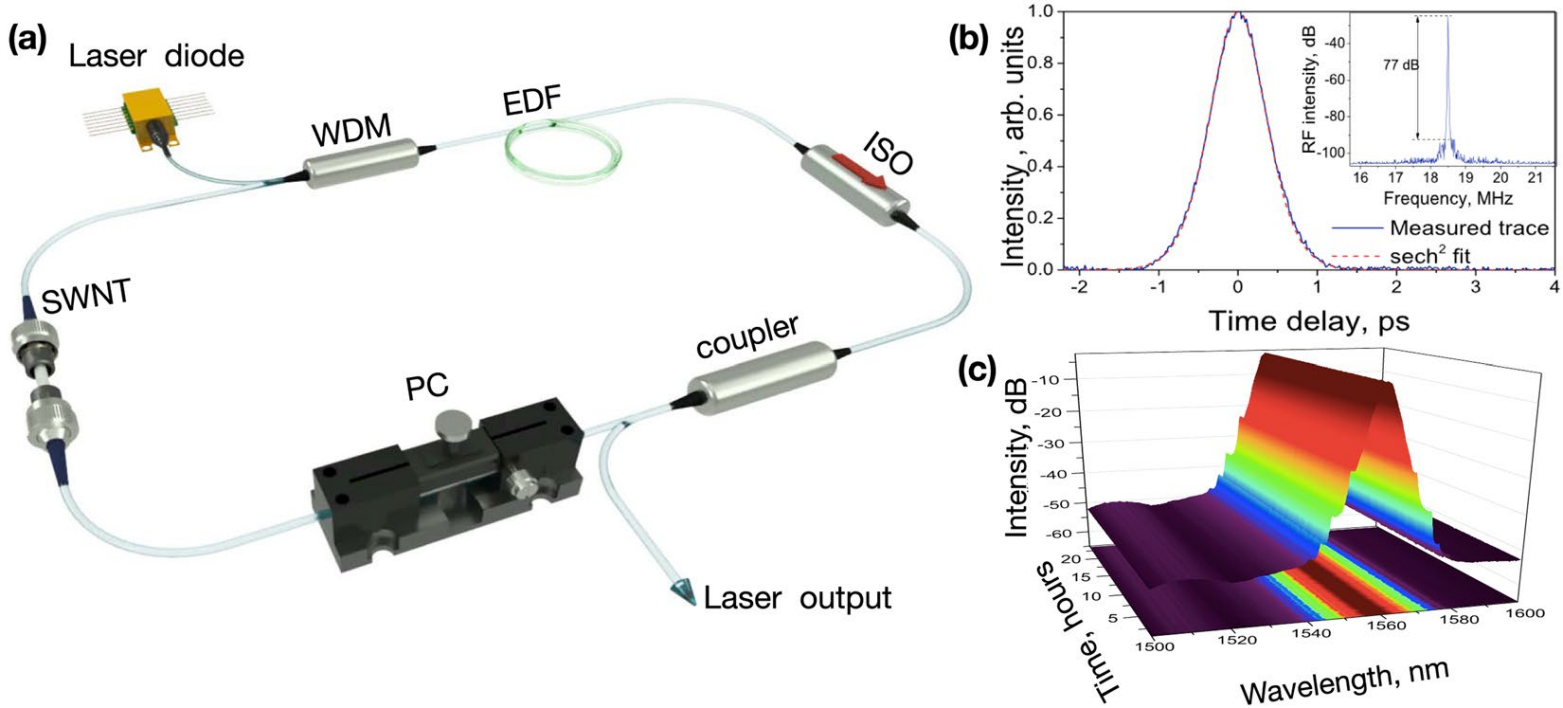
(**a**) Schematic setup of the ring Erbium-doped SWNT mode-locked fibre laser and its output parameters (**b**) autocorrelation trace (inset). (**c**) Output spectrum evolution during 24-hour continuous operation. Inset: The radio-frequency spectrum at the fundamental frequency and recorded with 6 MHz span.

To eliminate the possibility of degradation of the host polymer matrix, we performed similar experiment of pure SDBS/PVA and PVA samples laser ablation with a optical fluence of 2.3 mJ · cm^−2^ for 2 hours. Examination of the PVA-SDBS composites using digital microscopy does not demonstrate any evidence of degradation.

We also performed measurement using scanning electron microscopy (SEM), where is was noted that the samples were too sensitive to the high energy electron beam and thus high resolution imaging was not possible (see Supplementary Fig. S2). SEM images reveal significant difference in the laser ablated and unexposed areas of the film.

### Spectroscopic analysis of the SWNT-PVA films after laser radiation exposure

The bottom of the craters formed on the SWNT-PVA films were probed by micro-Raman spectroscopy (Fig. 4) to analyse their chemical composition after laser irradiation. As the size of the interaction volume (∼1–2 and ∼3–5 *μ*m in the lateral and axial directions, respectively) investigated during micro-Raman spectroscopy analysis was commensurate with the dimensions of the as-produced craters, the analysis could be performed directly.

For samples exposed to low doses of irradiation or for short periods of time (up to 6 hours of continuous irradiation), there are no discernable differences in the Raman spectra collected from the exposed crater and unexposed areas, when the spectra are normalised to the intensity of the G band. As seen in Fig. 4(a), there are no changes in the intensity or the shape of the G band, commensurate with no evidence of charge doping or strain effects. The intensity of the D band (relative to the G band) also remains unchanged, as I_*D*_:I_*G*_ is ∼0.04 in all analysed craters. Thus, the ultrashort pulsed laser irradiation does not induce any measurable structural changes to the nanotubes under examination, either as damage or chemical functionalisation.

It is interesting to note that the increasing crater depth (and thus decreasing total film thickness) afforded after extensive and high power laser radiation could also be confirmed by micro-Raman spectroscopy. In general, the intensity of the G band (normalised to acquisition time and conditions) was found to be lower at the bottom of the crater than on the top surface (see Fig. 4b). For example, after 10 minutes of laser irradiation just above the pump threshold (with powers lower than 5 mW), the intensity of the G band at the bottom of the crater is 81.6% of the intensity measured on the top unaffected surface. With the laser power fixed at 50 mW and an increase of laser exposure duration, the intensity of the G band decreases further: 66.2% – for 2 hours irradiation at 50 mW, 52.7% – for 6 hours at 50 mW and 5.4% – for 24 hours operation at 50 mW. Furthermore, after the 24 hours operation, weak photoluminescence features at ∼1400 cm^−1^ associated with the glass slide upon which the samples were mounted appeared (Fig. 4a), providing further evidence of decreasing film thickness.

Under the most extreme laser conditions, *i.e*. after 40 hours of continuous irradiation with 50 mW average power, the contribution from fluorescence becomes more pronounced (Fig. 4a). It is important to note that this is broadband photoluminescence, inconsistent with that expected from glass and thus suggests that these conditions facilitate the greatest changes in the structure and composition of the film. Although the substantial impact of photoluminescence complicates analysis, the retention of a low ratio of D and G bands intensities (I_*D*_:I_*G*_) and no changes in the positions of other vibrational modes imply that the nanotubes are not affected by this extreme laser exposure. Furthermore, it is not possible to directly appraise changes in the Raman spectra of the host polymer due to the resonance effect which preferentially enhances the bands associated with SWNTs. However, the presence of broadband fluorescence covering a wide range of energies would suggest that the intensive ultrashort pulsed radiation has resulted in a change to the structure of the polymer. We presumably associate this with the formation of polyenes and/or polycyclic aromatic hydrocarbons (PAHs) which arise under thermal (laser-assisted) degradation of PVA or SDBS^34^. Origination of PAHs on the crater surface forms a capping layer, which is robust to further laser radiation exposure.

PAH formation was expected to be observed by X-ray photoelectron spectroscopy (XPS) (see Supplementary Fig. S1), however, the signal from SWNT-PVA film was obscured by signal of index matching gel, used to fix films on the edge of fibre ferrule.

## Discussion

Even though the we observed a change in the structure of the surface after high-power laser radiation, the polymer (PVA) and the polymer/surfactant (PVA-SDBS) composites with dispersed SWNTs show high stability. Meanwhile, the pure composites without SWNTs do not undergo significant structural changes. This observation agrees with reports concerning the very high thermal conductivity of carbon nanotubes^35,36^. Indeed, isolated SWNTs possess extremely high longitudinal thermal conductivity, reaching 2800–6000 W · m^−1^ K^−1^ ^37^. Thermal conduction by carbon nanotubes is governed by the coupled vibrations of carbon atoms in the graphitic lattice and therefore can be referred to as a phonon conductivity^31,38^ with the mean free path for phonons in SWNTs around the hundreds of nanometers scale^39^. Due to this fact, SWNT composites have recently attracted significant research interest in their technological applications as a “heat sink” in electronics and aerospace industries. The thermal properties have been experimentally and theoretically investigated as suspensions, composites and at single nanotube levels^40,41^. Without dispersed SWNT, the PVA control composite is almost transparent for Vis-IR laser radiation (Fig. 1) and thermally insulating, since the polymer matrix features a small mean free path for phonons of several angstroms^42^.

Intuitively, the depression and crater formation observed could be attributed to localised melting and subsequent evaporation caused by laser exposure at sites containing a critical level of SWNT aggregates. Under the laser radiation exposure, SWNTs conduct the majority of the absorbed heat power to the polymer host matrix. Good physical contact and, therefore, interfacial thermal resistance, between SWNT and PVA allows relatively high efficiency in thermal energy transfer. In air at temperatures higher than 180–190 °C PVA irreversibly decomposes, undergoing pyrolysis and producing ethanolic acid (C_2_H_4_O_2_) and formaldehyde (CH_2_O(H-CHO)) with the increased volumes of CO and CO_2_ gases. The most important process of degradation is the elimination of water, similar to the process which has been shown to take place in the air at 150–250 °C^43^.

In conclusion, we have shown results on the surface modification of SWNT-PVA film samples upon high power femtosecond laser ablation. The order of magnitude considerations in this paper could be refined considerably by a quantitative analysis of the electromagnetic field of laser radiation, the thermal field, and stress field configuration near high concentration and big bundles of SWNT in the PVA matrix. However, because of the unordered arrangement of SWNTs in the sample, such refined calculations, although desirable, are not essential. The resulting conclusions may be drawn from our qualitative investigation: (1) The PVA-SDBS composite without SWNTs does not undergo modifications at the highest available laser powers and energies. SWNTs feature high heat conductivity; therefore, the PVA-SDBS matrix hosting SWNTs shows surface alteration at quite low laser power and on a short time scale. The high concentration of SWNTs and bundles of SWNTs leads to local heat transfer from the laser beam to the polymer and results in slight alterations in the geometries of the formed craters. (2) The most important process of structural alteration observed is the elimination of water together with the PVA-SDBS matrix decomposition. (3) A PAH capping layer, formed by laser irradiation of the polymer host matrix, changes the composites saturable absorption properties, leading to the introduction of additional reflection and scattering on roughness and SWNT bundles of large diameters formed close to the surface. More importantly, it improves the overall thermal stability and helps to sustain a laser optical fluency of 2.3 mJ · cm^−2^. The polymer samples with dispersed SWNTs demonstrate stabilisation of the resulted crater. We would like to stress, that the laser cavity configuration optimisation also plays a critical role in SA stability, as rapid alterations of the intracavity power and switching between mode-locking and Q-switched regimes causes irreversible damage to the sample, despite the power of the radiation.

We believe that results we report here justify that an SWNTs dispersed in a PVA-SDBS polymer matrix form a reliable saturable absorber. The laser treatment forming a capping layer even at lower laser intracavity powers is beneficial regarding improving the thermal robustness of SWNT-based SAs. It suggests a consistent solution for industrial fabrication of such SAs for commercial high-power and high-energy ultrafast lasers.

## Methods

### Mode-locked fibre laser design

The schematic of the proposed design of the laser system is presented in Fig. 5(a). The ring laser cavity consists of a 2 m long erbium-doped fibre (EDF) Liekki Er30-4/125, a 3-dB output coupler, in-line fibre isolator, and a polarisation controller (PC). All optical components are pigtailed with standard SMF-28 fibre. The SWNT-PVA film SA is placed after the output coupler to reduce the saturation of the device. The laser is pumped via a fibre Bragg grating stabilised laser diode operating at 976 nm with the maximum pump power of 600 mW through the isolating wavelength division multiplexor (WDM).

The total cavity length is ∼10.8 m. The laser mode-locking threshold pump power is 80 mW. At the maximum available pump power of 600 mW, the measured average output laser power reached is 48.7 mW. Figure 5(b) shows the autocorrelation trace, which allows estimating pulse duration as 550 fs, assuming sech^2^ profile. The laser repetition rate is 18.5 MHz (see inset in Fig. 5(b)). The inset in Fig. 5(b) demonstrates the high electric signal-to-noise ratio (SNR) of the fundamental frequency of 77 dB. The RF spectrum does not show the generation of sub-harmonics and justifies single pulse mode-locking.

For the stability check, the laser has been set for free-running for 24 hours under the laboratory conditions at a pump power of 600 mW. The pulse energy reached 2.63 nJ, with corresponding peak power of 4.78 kW. Figure 5(c) presents the evolution of output spectrum with a recording interval of 10 min during the laser operation. During 24 hours continuous operation, there is no noticeable spectral change. However, during the ongoing running of the laser, the output power decreased almost linearly from 49.3 mW to 45.6 mW. In 24 hours laser was switched off. After several on-off cycles, the same sample ensured stable self-starting mode-locking regime with the help of slight PC tuning.

### SWNT-PVA film preparation and characterisation

#### SWNT-PVA film synthesis

Commercially available purified SWNTs (2 mg, Unidym) grown by high-pressure CO conversion (HiPCO) were placed in deionised water (10 ml) containing SDBS (10 mg, Sigma-Aldrich). The dispersion then was subjected to sonication using a commercial ultrasonic processor (Nanoruptor, Diagenode) for 1 hour at 200 W and 20 kHz^44^. The suspension was processed by ultracentrifugation (Optima Max-XP ultracentrifuge, Beckman Coulter) with MLS-50 rotor at 25000 rpm for 1 hour, removing residual SWNT large bundles and impurities. The resulting dispersion was mixed with PVA powder (1 g, from Wako Pure Chemical Ind. Ltd., Japan) and placed in the Petri dish. A SWNT-PVA film was then obtained by drying the sample in the desiccator for a few days.

#### SWNT-PVA film characterisation

Photoluminescence: A HORIBA Jobin Yvon excitation emission spectrofluorometer (Fluorolog-3) was used to measure the PL map. The system is equipped with a Xenon lamp excitation source, and an InGaAs array matrix detector (Symphony Solo) cooled down by liquid nitrogen. PL maps are recorded by scanning the excitation wavelength from 300 to 800 nm with 5 nm steps and an exposure time of 30 s at an emission range of 950 to 1550 nm. In all the measurements, the entrance and exit slit widths are 14 nm.

Optical microscopy: We used a VHX5000 digital microscope (Keyence) and the VH-Z600T lens with 500–5000x magnification and numerical aperture N_*A*_ = 0.82. The average magnification used is 2000x which allowed better resolution. The lens is equipped with coaxial vertical illumination adapter with half mirror, aligning the axis of the incident light with the optical axis of the lens. Such a configuration allows an increase in the amount of the regular reflection from the investigated sample and, therefore, bright-field observation. The depth profile measurements are performed with z-axis resolution of 0.1 *μ*m.

Micro-Raman spectroscopy: A Horiba-Jobin-Yvon LabRAM HR spectrometer was used for Raman spectroscopy. Spectra were recorded using a 785 nm laser at 2.5 mW power, a 100x objective and a 50 *μ*m confocal pinhole to improve the spatial resolution in the z-direction to ∼3–5 *μ*m. To scan a range of Raman shifts simultaneously, 600 lines · mm^−1^ rotatable diffraction grating along a path length of 800 mm was employed. Spectra were detected by a Synapse CCD detector (1024 pixels) thermoelectrically cooled to −60 °C. Before measurements, the instrument was calibrated using the Rayleigh line at 0 cm^−1^ and a standard Si(100) reference band at 520.7 cm^−1^. A spectrum was recorded by averaging 8 acquisitions of 5 s duration.

Power-dependence measurements: To characterise the power-dependent absorption and modulation depth of the SA, a home-made Er-doped laser generating 560-fs pulses at 18.5 MHz repetition rate with 7.16 mW average output power was used as the probe. The probe laser output power was varied via a fibre attenuator while the rest laser output parameters were strictly fixed. The output was then followed by a 3-dB optical coupler from which one arm provides radiation on SWNT-PVA film sample, and the other arm serves as a reference. During the measurement, the power of the probe, radiating on the SWNT-PVA film sample, increased from 0.1 to 3 mW. The sample absorption decreases with the peak power increase according to^45^:

1 $$\alpha (I)=\frac{{\alpha }_{0}}{1+\frac{I}{{I}_{sat}}}+{\alpha }_{ns}$$

here *α*_0_ - is modulation depth of the SA; *α*_*ns*_ denotes non-saturable losses; *I* indicates launched peak intensity; *I*_*sat*_ is the saturation intensity.

## Electronic supplementary material

Supplementary material
