# Supplementary material for: Revealing the nature of morphological changes in carbon nanotube-polymer saturable absorber under high-power laser irradiation
Source: Sci Rep. 2018 May 10;8:7491. doi: 10.1038/s41598-018-24734-z (PMC5945804; doi:10.1038/s41598-018-24734-z)
Supplement: Supplementary file 1 — Supplementary material [file 41598_2018_24734_MOESM1_ESM.pdf]

Supplementary material
Revealing the nature of morphological changes in carbon nanotube-polymer saturable absorber under high-power laser irradiation

Maria Chernysheva[1,*], Mohammed Al Araimi[1,2], Graham A. Rance[3], Nicola J. Weston[3], Baogui Shi[4], Sayah Saied[4], John L.Sullivan[4], Nicholas Marsh[5], and Aleksey Rozhin[1]

[1]Nanotechnology Research Group and Aston Institute of Photonic Technologies, Aston University, Birmingham, B4 7ET, UK
[2]Al Musanna College of Technology, Muladdah, Al Musanna, Sultanate of Oman
[3]Nanoscale and Microscale Research Centre (nmRC), Cripps South, University of Nottingham, University Park, Nottingham, NG7 2RD, UK
[4]Surface Science Research Group, Aston University, Birmingham, B4 7ET, UK
[5]Keyence(UK), Ltd, Avebury House, 219-225 Avebury Boulevard, Milton Keynes, MK9 1AU, UK
[*]m.chernysheva@aston.ac.uk

X-ray photoelectron spectroscopy (XPS)

X-ray photoelectron spectroscopy was conducted using a Thermofisher ESCALAB 250 electron spectrometer equipped with a hemispherical sector energy analyser. A monochromated Al K_α X-ray source was used for analysis at a source excitation

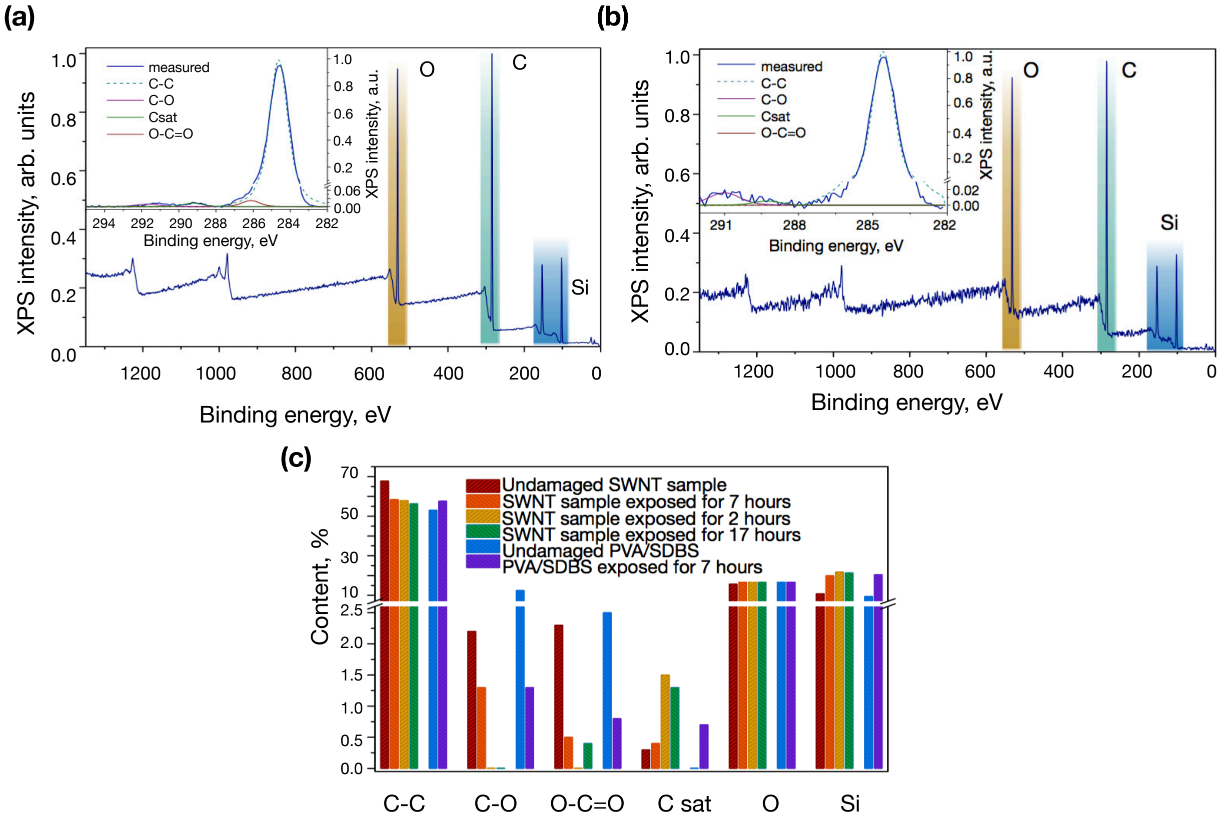

Figure S1. XPS spectrum of SWNT-PVA sample a) before and b) after laser irradiation with a power density of 0.1 MW·cm^{-2} for 17 hours; c) Statistical analysis of the composition of laser affected and unaffected SWNT-PVA and pure PVA samples.

energy of 15 KeV and emission current of 6 mA. The analyser pass energy was 20 eV with a step size of 0.1 eV and dwell time of 50 ms throughout the experiments. The base pressure within the spectrometer during examinations has been preserved at $5{\cdot}10^{-10}$ mbar level. This value ensured that all signals recorded are from the sample surface and no contamination has been introduced from the vacuum chamber. The resolution of the instrument is better than 0.4 eV. Curve synthesis was conducted using software embedded in the instruments Avantage data system. Analyses were conducted using an X-ray spot size of 120 μm and, where appropriate, the monochromated X-ray beam is positioned at the centre of a crater. Relative atomic concentrations were calculated from the intensities of the primary photoelectron spectral lines utilising codes incorporated in the instrument data system using Schofield cross sections.

For XPS analysis the size of the X-ray spot used during analysis is 120 μm in diameter, which is much larger than a typical crater. Therefore, the beam spot size was increased up to 250 μm to produce larger crater diameters, whilst preserving the launched optical fluence. The other set of laser ablated SWNT samples was produced at 20 $nJ{\cdot}cm^{-2}$ and a power density of 0.1 $MW{\cdot}cm^{-2}$ during a maximum of 17 hours.

During XPS analysis we investigated the region corresponding to carbon and its carbon-oxygen and carbon-carbon compounds (Fig. S1a), particularly C-C (with corresponding C 1s binding energy of 284.6 eV), C-O (286.1 eV), C=O (287.6 eV) and O-C=O (288.9 eV). The presence of C-O and O-C=O bonds are due to residual acetate (ester) groups (-O-C=O-CH_3). During PVA production, vinyl acetate polymer is hydrolysed in the presence of catalysts to replace acetate groups by hydroxyl groups (-OH). However, by controlling the degree of hydrolysis, *i.e.* setting an amount of acetate groups, one can control water penetration in the resulting PVA matrix[1]. The PVA used has 86.5-89% degree of hydrolysis, which is consistent with XPS measurements. The C 1s satellite peak shows the presence of a carbon ring structure. Si is present as an organic Si – siloxane, which contamination we refer to index matching gel application, to fix the sample between optical fibre connectors. Therefore, its variation can be neglected in the analysis.

The XPS spectra of unaffected samples and samples after laser radiation exposure were compared. The polymer composite and sample with dispersed SWNTs have demonstrated a similar trend of modification. We can conclude that laser modified samples (both SWNT composites and PVA-SDBS) feature significant decreases of the C-O and O-C=O components; as well as an increase of carbon ring structures. However, in the case of SWNT composite one can observe a decrease of C-C component, while in PVA-SDBS sample it increases. No consistent variation of the carbon-oxygen compounds and overall surface composition after high-power laser exposure with a change of power and duration of irradiation has been observed.

The increase of C-satellite intensity in the XPS spectra together with broad photoluminescence discovered in the Raman spectrum of the laser ablated area of the SWNT-PVA sample both justify the formation of polycyclic aromatic hydrocarbons, acting as a robust laser irradiation coating for the SWNT-PVA. The formation of such a coating stabilises the depth and width of the crater during laser exposure.

Unfortunately majority of the signal from SWNT-PVA sample was overshadowed by signal from the index-matching gel, which was used for attaching polymer film onto fibre ferrule edge. XPS demonstrates main features of index gel XPS of organic siloxane at 102 (Si2p), 154 eV (Si2s), carbon at 285 eV and oxygen at 532 eV.Thus XPS cannot provide detailed information on the structural changes on the films surface.

Scanning electron microscopy (SEM)

To perform SEM measurements, samples were coated with a 4nm layer of Ir and analysed using SEM (JEOL 7100F). The samples are quite sensitive to the electron beam. Figure S2 shows clear "burn" mark around the crater as a consequence of imaging at high voltage. Therefore, to prevent film destruction, the energy of the electron beam has been decreased down to 1.5 kV, thus, the sample seemed more stable. Though the resolution of the measurements was not enough due to low energy

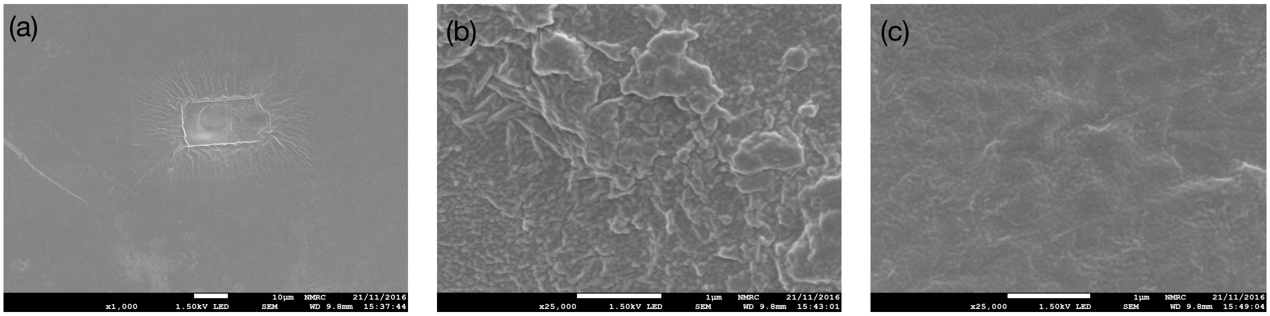

Figure S2. SEM image of SWNT-PVA sample: a) with the x1000 magnification; b-c) with x25000 magnification ofcrater bottom (b) and unexposed area (c).

of the electron beam, the apparent difference in the morphology at the bottom of the crater relative to the top surface can be observed. The top unexposed surface appearing largely featureless (or with some large features Fig. S2c), whereas the bottom of the crater has lots of small-domain size features (Fig. S2b). We cannot say definitively what these features are, but as we do not see any evidence of nanotubes, the observed change in morphology must be related to changes in the polymer (potentially from dehydration, cross-linking, forming PAHs and other carbon-rich structures).

References

1. Hallensleben, M. L. *Ullmann's Encyclopedia of Industrial Chemistry* (Wiley-VCH Verlag GmbH Co. KGaA, 2000).
